
JOURNAL INFORMATION
==============================
Journal ID: Wirtschaftsdienst

Wirtschaftsdienst

EISSN: 1613-978X

ARTICLE INFORMATION
==============================
PMCID: PMC9708501
DOI: 10.1007/s10273-022-3328-8
Article ID: 3328
Article version: 1
Subjects: Ökonomische Trends

Revisionen in den Volkswirtschaftlichen Gesamtrechnungen in Zeiten von Corona

Revisions in National Accounts in Times of Corona

Janz Christian Christian.Janz@destatis.de
1
Kuntze Peter Peter.Kuntze@destatis.de
2
Mucha Tanja Tanja.Mucha@destatis.de
3
1 grid.432326.2 0000 0001 1482 0825 Referat D12 Private Konsumausgaben, Statistisches Bundesamt, Gustav-Stresemann-Ring 11, 65189 Wiesbaden, Deutschland
2 grid.432326.2 0000 0001 1482 0825 Referat D11 Entstehung des Inlandsprodukts, Statistisches Bundesamt, Gustav-Stresemann-Ring 11, 65189 Wiesbaden, Deutschland
3 grid.432326.2 0000 0001 1482 0825 Referat D13 Koordinierung und Analyse des BIP, Statistisches Bundesamt, Gustav-Stresemann-Ring 11, 65189 Wiesbaden, Deutschland
Electronic publication date: 2022 Nov 30
Print publication date: 2022
Volume: 102
Issue: 11
First page: 904
Last page: 906
Copyright: © Der/die Autor:in 2022
License: Open Access: Dieser Artikel wird unter der Creative Commons Namensnennung 4.0 International Lizenz veröffentlicht (https://creativecommons.org/licenses/by/4.0/deed.de).
Open Access wird durch die ZBW — Leibniz-Informationszentrum Wirtschaft gefördert.
License URL: https://creativecommons.org/licenses/by/4.0/

Keywords: E01
issue-copyright-statement: © The Author(s) 2022

==============================

Die Volkswirtschaftlichen Gesamtrechnungen (VGR) stellen Politik, Wirtschaft, Wissenschaft und Öffentlichkeit ein detailliertes Abbild des wirtschaftlichen Geschehens in Deutschland zur Verfügung. Im Zentrum steht dabei das Bruttoinlandsprodukt (BIP), dessen preisbereinigte Veränderungsrate als wichtiges Maß für die konjunkturelle Entwicklung gilt. Bei der Erstellung der VGR bewegt sich die amtliche Statistik stets im Spannungsfeld zwischen Aktualität und Genauigkeit (Statistisches Bundesamt, 2021). Erste Informationen zum Wirtschaftswachstum sollen möglichst früh zur Verfügung gestellt werden – dies geschieht bereits 30 Tage nach Ablauf eines Quartals für das vierteljährliche BIP (Ackermann et al., 2021). Das erste Jahresergebnis wird spätestens 15 Tage nach Ablauf eines Jahres bekanntgegeben. Der Preis für diese frühen Veröffentlichungen ist eine noch nicht ganz vollständige Datengrundlage. Die Ergebnisse der VGR sollen jedoch nicht nur schnell, sondern auch hinreichend genau und detailliert bereitgestellt werden. Daher werden mit jedem Veröffentlichungstermin neue statistische Ausgangsdaten für zurückliegende Berichtszeiträume in die Berechnungen eingearbeitet und so die Qualität stetig verbessert.

Diese Überarbeitungen, sogenannte Revisionen, folgen einem festgelegten Rhythmus. Der Überarbeitungszeitraum variiert dabei je nach Termin zwischen wenigen Quartalen und vielen Jahren. Letzteres findet im Rahmen von Generalrevisionen in etwa fünfjährigen Abständen statt und dient der Integration neuer internationaler Konzepte, Klassifikationen, Datenquellen oder Berechnungsmethoden. Um keine Brüche in den Zeitreihen zu verursachen, werden dabei komplette Zeitreihen, zumeist bis zum Zeitpunkt der deutschen Wiedervereinigung, überarbeitet und zurückgerechnet. Die letzte Generalrevision fand 2019 statt und war eine überwiegend datenbedingte Revision, um neue bisher nicht verwendete Datenquellen zu integrieren. Die nächste Generalrevision ist europaweit harmonisiert und wird voraussichtlich 2024 durchgeführt (Statistisches Bundesamt, 2022a).

Eine jährlich wiederkehrende Aufgabe ist dagegen die Überarbeitung der vergangenen vier Jahre und der dazugehörigen Quartale in jedem Sommer – in diesem Jahr also die Ergebnisse der Jahre 2018 bis 2021. Größere Revisionen ergeben sich typischerweise vor allem für das Berichtsjahr t–2, also jetzt das Jahr 2020, für das zu diesem Zeitpunkt erstmalig die jährlichen Strukturstatistiken und weitere Jahresstatistiken vorliegen. Sowohl auf der Entstehungs- als auch auf der Verwendungsseite des BIP ersetzen die neuen detaillierten Basisdaten viele bis dahin vorläufige, mit Indikatoren fortgeschriebene Ergebnisse. Die Strukturstatistiken gehen in ihrer Detailtiefe deutlich über die Darstellung von Umsatz- oder Produktionsentwicklungen in den Konjunkturstatistiken hinaus. Somit sind mit diesen detaillierteren Daten auch Aussagen über Kostenrelationen und die Entwicklung der Wertschöpfung möglich. Zudem liegen für das Berichtsjahr t–2 erstmals Deflatoren für die Preisbereinigung der BIP-Aggregate auf Basis aktueller Güterstrukturen aus der Input-Output-Rechnung vor. Auch hierdurch kommt es zu Revisionen.

Durch die höhere Unsicherheit bei den Schätzungen in den von der Coronapandemie betroffenen Jahren sind die Revisionen ab 2020 stärker ausgefallen als sonst. Tabelle 1 zeigt die Revisionen des nominalen und des preisbereinigten BIP als Ergebnis der diesjährigen Sommerüberarbeitung der VGR. Für 2018 und 2019 gab es wenig neue statistische Informationen, sodass die Revisionen überschaubar sind. Die BIP-Revision für 2020 ist dagegen mit preisbereinigt +0,9 Prozentpunkten bzw. nominal 1,0 Prozentpunkten überdurchschnittlich stark ausgefallen (Statistisches Bundesamt, 2022a). Der durch den Beginn der Coronapandemie verursachte konjunkturelle Einbruch fiel mit -3,7 % zum Vorjahr etwas schwächer aus als zunächst ermittelt. In absoluten Zahlen ausgedrückt bedeutet dies für 2020 ein um knapp 38 Mrd. Euro höheres BIP-Niveau. Da für das Berichtsjahr 2021 die jährlichen Strukturstatistiken noch nicht vorliegen, wurden die revidierten Vorjahreswerte mit den bereits vorliegenden Konjunkturindikatoren erneut fortgeschrieben. Das nun

Tabelle 1
**Bruttoinlandsprodukt**

| | Preisbereinigt | | | In jeweiligen Preisen | | | In jeweiligen Preisen | | |
|---|---|---|---|---|---|---|---|---|---|
| | Neues Ergebnis | Bisheriges Ergebnis | Differenz | Neues Ergebnis | Bisheriges Ergebnis | Differenz | Neues Ergebnis | Bisheriges Ergebnis | Differenz |
| | Veränderung gegenüber dem Vorjahr in % bzw. Prozentpunkten | | | Veränderung gegenüber dem Vorjahr in % bzw. Prozentpunkten | | | Mrd. Euro | | |
| 2018 | 1,0 | 1,1 | -0,1 | 3,0 | 3,1 | -0,1 | 3.365,450 | 3.367,860 | -2,41 |
| 2019 | 1,1 | 1,1 | 0 | 3,2 | 3,1 | 0,1 | 3.473,260 | 3.473,350 | -0,09 |
| 2020 | -3,7 | -4,6 | 0,9 | -2,0 | -3,0 | 1,0 | 3.405,430 | 3.367,560 | 37,87 |
| 2021 | 2,6 | 2,9 | -0,3 | 5,8 | 6,0 | -0,2 | 3.601,750 | 3.570,620 | 31,13 |

höhere Ausgangsniveau 2020 führte zu einem ähnlich großen Revisionsbedarf und einem gleichsam höheren Niveau bei kaum veränderten Wachstumsraten 2021.

Der Hauptgrund für die überdurchschnittlich starken Revisionen des BIP ab 2020 ist die Coronapandemie, die die amtliche Statistik vor große Herausforderungen gestellt hat. So waren die Schätzunsicherheiten bei den ersten, vorläufigen Berechnungen 2020 durch das plötzliche weltweite Auftreten der Pandemie besonders groß: Sowohl die gestörten globalen Lieferketten als auch die allgemeine Verunsicherung in der Bevölkerung und der Wirtschaft mussten in den Schätzungen berücksichtigt werden. Darüber hinaus machten die staatlichen Maßnahmen – einerseits Geschäftsschließungen, Ausgangs- und Reisebeschränkungen bis hin zu kompletten Lockdowns, andererseits Konjunkturpaket und Unterstützungsleistungen – die Schätzungen deutlich schwieriger als in „normalen“ und konjunkturell ruhigeren Zeiten. Mit den nun vorliegenden detaillierteren Daten konnten diese Unsicherheiten deutlich reduziert werden.

Ein Beispiel hierfür ist die Fortschreibung der Bruttowertschöpfung (BWS) der einzelnen Wirtschaftsbereiche auf der Entstehungsseite des BIP, deren Entwicklung bis zum Vorliegen der Strukturstatistiken typischerweise durch die Entwicklung von Produktion oder Umsätzen berechnet wird. Dies ist aber gleichbedeutend mit der Annahme, dass sich der Einsatz von Vorleistungen (z. B. Roh-, Hilfs- und Betriebsstoffe, Vorprodukte, gewerbliche Mieten oder Transportkosten) genauso entwickelt wie der Umfang der Produktion (fixe Vorleistungsquoten). Diese Annahme schien in der Coronakrise nicht mehr haltbar: Bei einbrechender Produktion, aber aufgrund von Fixkostenbestandteilen gleichzeitig wesentlich schwächer sinkenden Vorleistungen, müsste zumindest in den von der Pandemie am stärksten betroffenen Wirtschaftsbereichen die BWS stärker zurückgehen als die Produktion, so die Überlegung. Auf Basis der Verwendungstabelle der Input-Output-Rechnung, die eine Kombination von Produktionsbereichen und verwendeten Gütern enthält, wurden deshalb wirtschaftszweigspezifische Zuschläge auf die Vorleistungen modelliert, die ab dem zweiten Quartal 2020 über vier Quartale mit abnehmender Stärke in die kurzfristigen Schätzungen einbezogen wurden. Mit der Sommerüberarbeitung der VGR und der Integration der Strukturstatistiken für 2020 hat sich nun gezeigt, dass diese Annahme steigender statt fixer Vorleistungsquoten zwar in einigen Wirtschaftsbereichen – wie dem Gastgewerbe und Teilen des Verkehrs – durchaus gerechtfertigt waren. In großen Bereichen wie dem Verarbeitenden Gewerbe oder den Unternehmensdienstleistern, die zusammen knapp ein Drittel der gesamten BWS ausmachen, traf dies jedoch nicht zu. Dadurch wurde der Rückgang der BWS in diesen Bereichen zu stark dargestellt und musste auch insgesamt um 1 Prozentpunkt nach oben korrigiert werden (Statistisches Bundesamt, 2022b).

Aufwärtsrevisionen gab es auch in der Verwendungsrechnung, der zweiten Säule der Inlandsproduktberechnung in den deutschen VGR. Die Verwendungsseite ist definiert als die Summe aus den Konsumausgaben der privaten Haushalte und des Staates, den Bruttoinvestitionen und dem Außenbeitrag. Die Integration der aktuellen Jahresstatistiken für 2020 mit der Sommerüberarbeitung ersetzte auch hier die vorläufige indikatorgestützte Fortschreibung. Die aktualisierten Ergebnisse für die privaten Konsumausgaben liegen deutlich über dem bisherigen Niveau. Maßgeblich waren unter anderem neue Erkenntnisse für Beherbergungs- und Gaststättendienstleistungen, die besonders von den Corona-Schutzmaßnahmen betroffen waren. Während in einzelnen Monaten ein Beherbergungsverbot für Privatreisende galt und Restaurants ihren Betrieb einschränken mussten, führten die Lockerungen in den Sommermonaten offenbar zu stärkeren Nachholeffekten als bisher angenommen. Die Jahreserhebung im Handel lieferte neue Erkenntnisse zu den verkauften Produkten 2020. Hier zeigten sich die erwarteten coronabedingten Strukturverschiebungen in den abgesetzten Sortimenten: So wurde einerseits überdurchschnittlich viel Obst, Gemüse und Fleisch gekauft, während andererseits die Nachfrage nach Bekleidung und Schuhen kräftig zurückging. Jedoch fiel der Anstieg bzw. der Rückgang geringer aus als anfänglich berechnet. In der Summe führten auch hier die neuen Informationen zu höheren privaten Konsumausgaben.

Die Bauinvestitionen liefen im ersten Coronajahr 2020 sogar noch etwas besser als die Ergebnisse bisher gezeigt hatten (preisbereinigt +1,4 Prozentpunkte). Die Investitionen in sonstige Anlagen – vor allem Ausgaben für Forschung und Entwicklung – mussten dagegen für 2020 um über 4 Prozentpunkte nach unten korrigiert werden. Maßgeblich waren erstmals vorliegende Erhebungsergebnisse des Stifterverbands für die Deutsche Wissenschaft zu den Forschungsausgaben und Ergebnisse zu Softwarekäufen aus den Struktur- und Investitionserhebungen. Auch hier zeigt sich die erhöhte statistische Unsicherheit in Zeiten der Coronapandemie.

Auch die Konsumausgaben des Staates mussten revidiert werden. Nachdem internationale Gremien einheitliche Regeln zu pandemiebedingten Vorgängen vereinbarten, mussten einige Transaktionen im Bereich der Sozialversicherung neu zugeordnet werden. So handelt es sich etwa bei den Corona-Prämien und Erstattungen für pandemiebedingte Mindereinnahmen um sonstige Subventionen. In der gesetzlichen Krankenversicherung betrifft dies Ausgleichszahlungen für Vorsorge- und Rehabilitationseinrichtungen sowie Heilmittelerbringer, die nun nicht mehr bei den Vorleistungen, sondern bei den sonstigen Subventionen des Staats gebucht werden. Dagegen wurden die Corona-Testungen für Bürger:innen, die ab 2020 aus Mitteln des Gesundheitsfonds gezahlt wurden, von den Vorleistungen zu den sozialen Sachleistungen umgebucht. Weitere Umbuchungen von den sozialen Sachleistungen zu den monetären Sozialleistungen fanden für die Entlastungsleistungen bei der Tages- und Nachtpflege sowie der Kurzzeitpflege und erhöhte Beiträge zu Pflegestufen im Pflegebereich statt. In der Summe errechneten sich durch die Änderungen deutlich niedrigere soziale Sachleistungen und Vorleistungen der Sozialversicherung und somit auch geringere staatliche Konsumausgaben in den beiden Coronajahren. Die unterschiedliche Entwicklung zwischen nominalem und realem Staatskonsum 2020 und 2021 ist vor allem auf die Überarbeitung des Deflators für Krankenhausbehandlungen zurückzuführen. Die Trennung der Pflegepauschale von den Krankenhauskosten in den Fallpauschalen sowie bis vor kurzem fehlende Informationen zur Entwicklung der Fallzahlen während der Coronapandemie erschwerten die ursprünglichen Berechnungen. Durch die vollständigere Datenbasis sank der Deflator für Krankenhausbehandlungen 2020 und 2021 erheblich im Vergleich zum Datenstand vor der Sommerrevision. Der neue, niedrigere Deflator hatte zur Folge, dass die reale Entwicklung der Krankenhauskosten deutlich anstieg.

Der Außenbeitrag – also der Saldo zwischen den Exporten und Importen von Waren und Dienstleistungen – wurde im Rahmen der Sommerüberarbeitung ebenfalls angepasst. Anlass bilden turnusmäßige Revisionen der Außenhandelsstatistik sowie der Zahlungsbilanzstatistik der Deutschen Bundesbank, bei denen im Wesentlichen Nachmeldungen eingearbeitet werden. Für 2020 wurden zwar sowohl Waren- und Dienstleistungsexporte wie auch Waren- und Dienstleistungsimporte revidiert, unter dem Strich blieb der Außenbeitrag aber nahezu unverändert. Ein relativ neuer Anlass für Revisionen in den VGR resultiert aus der Arbeit der Large Cases Unit (LCU) innerhalb der deutschen amtlichen Statistik (Ahlborn et al., 2021; Hörner et al., 2022). Diese spezialisierte Organisationseinheit beobachtet und analysiert seit 2020 große, multinationale Unternehmensgruppen. Ihr Ziel ist dabei die Qualitätssicherung der VGR und der zugrunde liegenden Wirtschaftsstatistiken. Die LCU führt alle amtlichen statistischen Informationen wichtiger Unternehmensgruppen zusammen, analysiert diese gezielt auf Konsistenz und klärt Unstimmigkeiten im direkten Unternehmenskontakt auf. Aus den Analysen ergibt sich dann auch Änderungsbedarf für die VGR. Diese können teils bereits in den laufenden Jahresüberarbeitungen berücksichtigt werden, teils aber – wenn Korrekturen in einzelnen Wirtschaftsbereichen zu spürbaren Zeitreihenbrüchen führen würden und länger zurückliegende Zeiträume betroffen sind – werden diese erst im Rahmen der kommenden Generalrevision im Sommer 2024 umgesetzt.

Die Überarbeitung der Ergebnisse führte nicht nur beim BIP, sondern auch bei weiteren Kennziffern der VGR zu Revisionen (Statistisches Bundesamt, 2022b), insbesondere das Bruttonationaleinkommen (BNE), das Grundlage für die BNE-Eigenmittel ist. Diese sind die mit Abstand wichtigste Finanzierungsquelle des EU-Haushalts. Während das BIP durch die jüngsten Überarbeitungen für 2020 knapp 38 Mrd. Euro höher ausgewiesen wird, sind es beim BNE über 44 Mrd. Euro.

**Title:** ***Revisions in National Accounts in Times of Corona***

This article explains the purpose, reasons and size of revisions in German national accounts. It presents the results of the revision carried out in the summer of 2022 for the past four reporting years and describes special features, including those caused by the coronavirus pandemic.

Christian Janz leitet das Referat „Private Konsumausgaben“ im Statistischen Bundesamt in Wiesbaden.

Peter Kuntze leitet dort das Referat „Entstehung des Inlandsprodukts“.

Tanja Mucha leitet dort das Referat „Koordinierung und Analyse des BIP“.


Literatur

Ackermann, A., X. Dickopf und T. Mucha (2021), Flash und Nowcast: Schnellschätzungen des Bruttoinlandsprodukts in der Corona-Pandemie, WISTA — Wirtschaft und Statistik, 4.
Ahlborn, M., F. Draken und V. Schulz (2021), Qualitätssicherung in der amtlichen Statistik: Large Cases Unit, WISTA — Wirtschaft und Statistik, 2.
Hörner, N., M. Rotsche und J. Söngen (2022), Fortschritte der Large Cases Unit, WISTA — Wirtschaft und Statistik, 5.
Statistisches Bundesamt (2021), Bruttoinlandsprodukt für Deutschland 2020: Begleitmaterial zur Pressekonferenz am 14. Januar.
Statistisches Bundesamt (2022a), Qualitätsbericht Volkswirtschaftliche Gesamtrechnungen, 9.
Statistisches Bundesamt (2022b), Volkswirtschaftliche Gesamtrechnungen, Sommerrechnung 2022 — Revisionen und überarbeitete Ergebnisse.
